# *BMPR2* promoter methylation and its expression in valvular heart disease complicated with pulmonary artery hypertension

**DOI:** 10.18632/aging.203690

**Published:** 2021-11-18

**Authors:** Ni Li, Linwen Zhu, Caimin Zhu, Hua Zhou, Dawei Zheng, Guodong Xu, Huoshun Shi, Jianqing Gao, Albert Jiarui Li, Zhaoyang Wang, Lebo Sun, Xiajun Li, Guofeng Shao

**Affiliations:** 1 Department of Cardiothoracic Surgery, Lihuili Hospital Affiliated to Ningbo University, Ningbo City, Zhejiang 315041, China; 2 Institute of Pharmaceutics, College of Pharmaceutical Sciences, Zhejiang University, Hangzhou, China; 3 Edgemont High School, Scarsdale, NY 10583, USA; 4 School of Life Science and Technology, ShanghaiTech University, Shanghai 201210, China

**Keywords:** DNA methylation, valvular heart disease (VHD), *BMPR2*, pulmonary artery hypertension (PAH), biomarker, apoptosis

## Abstract

Valvular heart disease (VHD) is a common heart disease that affects blood flow. It usually requires heart surgery. Valvular heart disease complicated with pulmonary artery hypertension (VHD-PAH) may be lethal due to heart failure that results from increased heart burden. It is important for these patients to seek early treatment in order to minimize the heart damage. However, there is no reliable diagnosis method in VHD. In this study, we found DNA methylation was increased at the promoter of *BMPR2* gene in the VHD patients compared with the healthy controls. This finding was confirmed by an independent cohort study of VHD patients and healthy controls. In addition, *BMPR2* mRNA levels were reduced in the plasma of the VHD patients. There is strong correlation between *BMPR2* promoter DNA methylation and the severity of VHD. Indeed, we found that both *BMPR2* promoter DNA methylation and *BMPR2* mRNA levels in the plasma are good biomarkers of VHD by themselves, with the respective AUC value of 0.879 and 0.725, respectively. When they were used in combination, the diagnostic value was even better, with the AUC value of 0.93. Consistent with the results in the VHD patients, we observed decreased BMPR2 and increased fibrosis in the lung of a PAH model mouse. BMPR2 was also decreased in the hearts of the PAH mice, whereas BMP4 was increased. Furthermore, BMPR2 was reduced in the heart valve tissue samples of human VHD patients after valve replacement with moderate/severe PAH compared with those with mild PAH. There was also increased apoptosis in the hearts of the PAH mice. *BMPR2* promoter DNA methylation and its expression appear to be good biomarkers for VHD. Our results also suggest that DNA methylation may cause PAH through deregulation of BMP signaling and increased apoptosis.

## INTRODUCTION

A heart valve is a one-way valve located at both the entrance and exit of the heart, and it helps to maintain the one-way blood flow. There are four valves on the heart, with mitral valve (MV) located at the entrance and aortic valve (AV) located at the exit of the left ventricle, and tricuspid valve (TV) at the entrance and pulmonary valve (PV) at the exit of the right ventricle [1, 2]. Through the cooperative work of the ventricles and the valves, blood flows unidirectionally inside the blood vessel, which prevents backflow to minimize any useless heart work. Valvular heart disease (VHD) is a clinical problem that affects one or more of these valves so that they cannot function properly to maintain one-way blood flow and normal blood hemodynamics [3–6]. There are two main types of lesions. One is stenosis, which causes poor blood flow; the other is incomplete closure, which causes blood to flow backward [7]. Both of these lesions can lead to increased heart burden and insufficient blood flow, and advanced lesions can lead to heart failure [5, 8]. VHD is one of the main diseases requiring adult cardiac surgery which results in most cardiovascular morbidity and mortality in China [9, 10]. However, there is no reliable and effective early diagnosis method in VHD patients suffering from serious complications and irreversible valvular dysfunction. Valve surgery is considered to be one of the main treatment options. VHD does great harm to the health of patients and it can lead to many complications. For instance, VHD can lead to complications of pulmonary artery hypertension (VHD-PAH), which is even worse for the health of the patients [11]. It is critical for the VHD patients complicated with pulmonary artery hypertension to seek effective treatment as early as possible. Further understanding of its pathogenesis will help the doctors to make good judgment decisions in choosing the right treatment option for the valvular surgery.

In this study, we have analyzed many VHD-PAH patients, and examined if there was any correlation between DNA methylation levels and the risk of pulmonary artery hypertension in patients with valvular heart disease. VHD-PAH causes valve stenosis. It changes the volume load of pulmonary vessels and makes the pulmonary vessels flow continuously under high pressure. It results in intimal edema from the interstitial fluid because of high pulmonary vein pressure, which ultimately leads to structural change of the lung as well as respiratory dysfunction. This will increase the risk during surgery and may result in death in severe cases when the patient's pulmonary respiratory function deteriorates. Therefore, early detection is critical for targeted treatment of VHD-PAH patients, which might prevent further progression of the disease after the surgical treatment.

Recent developments in epigenetic techniques including DNA methylation analysis have provided novel early detection methods for many diseases [12–14]. The pathogenesis of VHD is complex and it is still not fully understood. Gene misexpression, inflammation and auto-immune responses may contribute to VHD-PAH pathogenesis and its complication [3]. In recent years, great efforts have been made to determine whether dysregulation of DNA methylation plays an important role in the occurrence and development of VHD. In our previous study, we found DNA methylation level of the brain natriuretic peptide (*BNP*) gene was associated with pathogenesis of rheumatic heart disease (RHD) which can also cause VHD [15, 16]. In this study, we tested *BMPR2* in the VHD-PAH patients as a new biomarker since it was reported that mutations in *BMPR2* cause PAH in the literature [17–19]. BMPR2 protein can cause excessive proliferation of pulmonary vascular endothelium and smooth muscle cells. Mutations in *BMPR2* are found in 55–70% of severe pulmonary arterial hypertension (HPH) patients and 11−40% of idiopathic pulmonary arterial hypertension (IPH) cases [7, 20–22]. Abnormal DNA methylation at the promoter region of a gene can lead to changes in the level of gene expression without changing the DNA sequence [23, 24]. It is also unclear whether pulmonary arterial systolic pressure (PASP) can cause genetic or epigenetic changes in VHD patients undergoing mechanical heart valve replacement, especially with different types of surgical operation, including mitral valve replacement (MVR), aortic valve replacement (AVR) and double valve replacement (DVR). Anticoagulation is a key treatment for patients with VHD after valve replacement. Interestingly, long-term anticoagulation therapy with warfarin can influence DNA methylation of the *BNP* gene in RHD patients undergoing mechanical heart valve replacement therapy reported in our previous studies [16, 25, 26].

The goal of this study is to assess whether DNA methylation at the *BMPR2* promoter and its expression may be associated with the increased risk of VHD-PAH. Indeed, hypermethylation of the *BMPR2* promoter occurs in the PAH patients and it is associated with reduced BMPR2 expression. Thus, we analyzed DNA methylation of the *BMPR2* promoter in the peripheral blood of VHD-PAH patients and healthy control people. We also quantified *BMPR2* mRNA levels in these samples. We examined the DNA methylation level at the promoter of the *BMPR2* gene in VHD patients after long-term administration of anticoagulation drug warfarin. In addition, we assessed the effects of other factors such as age and gender on DNA methylation of *BMPR2*. We generated a pulmonary artery hypertension (PAH) mouse model through abdominal shunt. Interestingly, we observed reduced BMPR2 expression in the lung or heart of the PAH model mice by immunostaining or western blot. Furthermore, there was increased BMP4 and apoptosis in the hearts of PAH mice. These results indicate that deregulation of BMP signaling is important for VHD-PAH and *BMPR2* promoter DNA methylation and mRNA levels can serve as good biomarkers for VHD-PAH.

## MATERIALS AND METHODS

### Patients and research subjects

The research subjects were selected from the patients in the inpatient clinic of Ningbo Medical Center of Li Huili Hospital in Ningbo City of Zhejiang Province in China between January 2017 and December 2018. The research subjects were comprised of 91 VHD-PAH patients and 64 healthy controls with no reported medical history of congenital heart disease, cardiomyopathy, liver or renal disease. Among them, 55 VHD-PAH patients and 28 controls were used for *BMPR2* DNA methylation analysis. A total of 36 VHD-PAH patients and 36 controls were used for *BMPR2* mRNA quantification. DNA methylation analysis was also performed for *BMPR2* in the second cohorts of 27 VHD-PAH patients and 29 healthy control people (see Supplementary Table 1). All experiments were carried out in accordance with relevant guidelines and regulations of Li Huili Hospital. The criteria used for the enrollment of the VHD patients were as follows: (i) diagnosis of mitral or aortic valve prolapse because of mitral or aortic chordae tendinae fracture and mitral or aortic insufficiency which requires mitral or aortic valve replacement; (ii) left ventricular ejection fraction (EF) > 50%; (iii) left ventricular end-diastolic diameter (LVEDD) < 55 mm. Pulmonary arterial pressure was measured by echocardiography and the patients were separated into two PAH groups: one with mild PAH condition, and the other with moderate or severe PAH condition, respectively. The patients are grouped as follows: mild PAH (30 mmHg ≤ PASP < 55 mmHg), and moderate or severe PAH (PASP ≥ 55 mmHg). The following case exclusion criteria were applied to the excluded patients: (i) those with lung disease; (ii) those with hyperthyroidism, hypothyroidism and chronic anemia; (iii) the patients with recent acute heart failure. The experiments on the human subjects were approved by the institutional oversight committee of Li Huili Hospital. The human patients were informed prior to this study and the written consent forms were obtained from the patients involved in this study. Different factors such as age, gender, smoking, etc. were included in *BMPR2* DNA methylation analysis which is summarized in Table 1.

**Table 1:** Characteristics of 55 VHD-PAH patients used for the *BMPR2* promoter DNA methylation analysis.

**Variable**	**Case (%)/mean ± SD**
Case number (*n*)	55
Age (year)	57.13 ± 1.487 (18–82)
Gender	
Male	26 (47.27%)
Female	29 (52.73%)
Cardiac function grading	
Grade II	7 (12.73%)
Grade III	43 (78.18%)
Grade IV	5 (9.09%)
Smoking	
Yes	7 (12.74%)
No	48 (87.26%)
Drinking	
Yes	28 (50.91%)
No	27 (49.09%)
Atrial fibrillation	
Yes	16 (29.09%)
No	39 (70.91%)
LVEF	0.60 ± 0.08 (0.35–0.78)
Operation type	
MVR	34 (61.82%)
AVR	11 (20%)
DVR	10 (18.18%)
mPAP (mmHg)	47.49 ± 2.682 (25–101)
Mild mPAP (*n* (%))	45 (81.82%)
Moderate/Severe mPAP (*n* (%))	10 (18.18%)

Abbreviations: mPAP: mean pulmonary artery pressure; LVEF: left ventricular ejection fraction; MVR: mitral valve replacement; AVR: aortic valve replacement; DVR: double valve replacement.

### Sample collection and DNA isolation

Blood samples were collected from the human subjects and immediately transferred into the tubes containing EDTA. Plasma was isolated after centrifugation of blood samples at 3000 rpm for 15 minutes and then the plasma samples were stored at −80°C. All blood samples of the patient cases and healthy controls were collected by the same investigators in the hospital. According to the manufacturer's instructions, we used QIAamp DNA Mini Kit (Qiagen, Hilden, Germany) to isolate genomic DNA from the plasma samples of 82 VHD-PAH patients and 57 controls. The quality and purity of the genomic DNA samples were measured by an ultra-micro nucleic acid UV tester (NanoDrop 1000, Wilmington, DE, USA).

### Bisulfite treatment

Genomic DNA samples were purified from the collected blood samples. Bisulfite treatment was performed with 500 ng of genomic DNA for each sample with the EpiTect^®^ DNA Bisulfite Kit (Qiagen, Germany) according to the manufacturer’s instructions.

### Pyrosequencing for measuring *BMPR2* DNA methylation levels

Polymerase chain reaction (PCR) of bisulfite-treated DNA sample was performed in the PCR reaction mixture containing 38 μl of PCR premix solution, 50 pmol of each primer and 2 μl of bisulfite-treated DNA sample. After forty PCR amplification cycles, the PCR product was subjected to pyrosequencing with the sequencing primer GTAGTGAGGGTTTTATT and the PyroMark Q96 System (Qiagen) according to the manufacturer’s instructions. In total, 10 CpGs of *BMPR2* were analyzed by the pyrosequencing detector (PyroMark Q96 ID) supplied with the Pyro Q-CpG software. The sequences of these *BMPR2* primers are provided as follows: the forward primer BMPR2-F has 5′-GGTGGGGTTTAGGAGTTTTTAGT; the reverse primer BMPR2-R contains 5′-ACCCTATCTATCTCTAACCTATAATAC; the sequencing primer BMPR2-S has 5′-GTAGTGAGGGTTTTATT. Biotin was used for 5′modification. The DNA methylation levels of 10 CpG sites of the VHD-PH cases, together with those of the controls, are shown in Supplementary Figure 1.

### Total RNA extraction

Total RNA in plasma was extracted using TRIzol LS reagent (Invitrogen, Karlsruhe, Germany) following the manufacturer’s instructions. The SmartSpec Plus spectrophotometer (BioRad, Hercules, CA, USA) was used to determine the concentration and purity of the purified RNA samples. The purified total RNA samples were stored at −80°C. The RNA samples were considered pure enough if their A260/A280 ratios ranged from 1.8 to 2.1, and then they were used for quantitative RT-PCR (qRT-PCR) analysis.

### Reverse transcription

The cDNA was synthesized from the purified RNA samples with random primers and oligo-dT primers included in the GoScript RT System (Promega) based on the manufacturer's instructions.

### Quantitative real-time PCR

*BMPR2* mRNA expression levels were measured by qRT-PCR using the GoTaq qPCR Master Mix (Promega) on a Mx3005P real-time PCR system (Stratagene, CA, USA). The specific primer sequences used for qRT-PCR analysis of the *BMPR2* mRNA are 5′-TGGCAAATCAGGATCAGGTG and 5′-CCAGCGATTCAGTGGAGATG. The sequences of the specific primers used for the reference glyceraldehyde 3-phosphate dehydrogenase (GAPDH) gene are 5′-ACCCACTCCTCCACCTTTGAC and 5′-TGTTGCTGTAGCCAAATTCGTT. The specificity of the qRT-PCR reaction was assessed by the melting curve with a single peak in the PCR product. The levels of *BMPR2* mRNA were calculated based on the ΔC_q_ method. The lower the ΔC_q_ value is, the higher the *BMPR2* mRNA level is [27, 28]. Relative *BMPR2* expression levels across different samples were calculated by using the 2^−ΔΔCq^ method [29]. All qRT-PCR experiments were repeated for three times, with *GAPDH* as the internal control.

### Establishment of pulmonary artery hypertension (PAH) mouse model by intraperitoneal shunt

The animal protocol for this study was approved by the Institutional Animal Care and Use Committee (IACUC) of Ningbo University in China, following similar international guidelines for the Care and Use of Laboratory Animals. To establish a mouse model for pulmonary artery hypertension, five C57BL/6 male mice weighing 20–30 g were anesthetized through intraperitoneal injection of 1% of pentobarbital sodium at 80 mg/kg before the abdominal median incision were made for these mice [30]. The intestinal tube of mice was pushed aside with cotton swab on the paved disinfection gauze so that the abdominal aorta and inferior vena cava were exposed for surgery after separation of the retroperitoneal membrane. The branch vessels were ligated under the microscope, and a 6-0 polyester line was inserted at the initiation of the left renal artery to block blood flow, just above the main iliac artery. A cut of about 0.3 mm was made for the anterior wall of the abdominal aorta with elbow microshear to expose the opposite lateral wall of the abdominal aorta. Then a longitudinal incision was made to connect the abdominal aorta with the inferior vena cava, with the incision length equal to half of the internal diameter of the abdominal aorta. The incision of the anterior wall of the abdominal aorta was continuously sutured with 11–0 polyacrylamide filaments. The blocking was loosened and the compression hemostasis was performed for 30 seconds to allow red blood to flow past the proximal end of inferior vena cava until obvious pulsation and swelling appeared. The mice were put back into a single mouse cage after 1 hour of recovery from the surgery. After 8 weeks, a pulmonary artery hypertension (PAH) mouse model was established and these PAH mice were used for experiments in this study. At the same time, 5 mice without any abdominal shunt were used as the controls. Pulmonary artery pressure (PAP) of these mice was measured at the end of 8 weeks. They were anesthetized before their trachea was cut for measuring the blood pressure and were fixed in the supine position. The mice were connected to the small animal ventilator at 120 times/min, with the tidal volume of 0.15 ml. The chest of the mice was cut open quickly, and their pericardium was sliced to expose the right ventricle. A polyethylene catheter filled with heparin sodium solution was applied to the PAH mice afterwards. The pressure of the PAH mice was recorded by a pressure transducer (Yilian, Shanghai, China) connected to one end of the catheter, with a 27G needle connected at the other head end of the catheter. The PAP values of these mice were recorded after their pressure waveform became stable. The mice were anesthetized before their tissue samples were collected for the experiments after euthanasia. Finally, the cardiopulmonary tissue was obtained and right ventricular hypertrophy index (RVHI) was measured with the following protocols. The chest of the dead mouse was quickly cut off to remove the entire heart and lung together. Then the needle was inserted from the right ventricle, and the blood was rinsed with saline until the lungs of the mouse were completely white. Both mouse lungs were soaked and fixed in 4% paraformaldehyde solution for HE and VG staining, respectively. Right ventricle interval and left ventricle interval were isolated simultaneously. The atrium and the large vascular root were cut along the atrial groove, whereas the right ventricular free wall was cut along the posterior interventricular wall groove. After the right ventricular free wall (RV) and left ventricle with ventricular interval (LV+S) were vacuated first and then weighed with precision electronic balance, RVHI is the weight ratio of right ventricle (RV)/left ventricle with ventricular interval (LV+S).

### Immunohistochemistry (IHC)

Paraffin sections of the lung and heart tissues of the PAH mice and control mice were baked for 40 min, and placed into the reagent tank for dewaxing, hydration, and incubation with 3% of hydrogen peroxide (diluted with pure water) for 10 minutes to eliminate the activity of endogenous peroxidase. Paraffin sections were then deparaffinized and rehydrated. Antigen retrieval was performed using heat-incubation in epitope retrieval solution (IHC World, USA) for 30 minutes. The primary antibodies used for immunohistochemistry were anti-BMPR2 (1:200) and anti-BMP4 (1:50) antibodies from Cell Signaling Technology, USA.

### Hematoxylin-Eosin (H&E) staining

Paraffin-sections were baked for 40 min, and placed into the reagent tank for dewaxing. They were stained with hematoxylin for 4 minutes and washed with water before they were subjected to differentiation with alcohol hydrochloride solution for 2 seconds followed by eosin staining for 2 minutes. Then they were dehydrated in 70% of ethanol for 5 seconds, 95% of ethanol for 5 seconds, and twice in absolute ethanol for 2 mins. The dehydrated sections were incubated in xylene twice with five minutes each. The sections were sealed with neutral gum.

### Van Gieson (VG) staining

Paraffin sections were subjected to dewaxing and dehydration as above. Then these dehydrated sections were stained with Weigert iron hematoxylin solution for 4 minutes. After hematoxylin was washed away briefly with water, the sections were incubated with the acid differentiation solution containing alcohol and hydrochloride for several seconds before being washed away for 2 minutes with water. The sections were stained with prepared VG dye solution for 2 minutes. The sections were dried with filter paper and quickly differentiated with alcohol and hydrochloride solution before they were dehydrated directly with 95% of ethanol. After they were dehydrated with anhydrous ethanol, the sections were treated with transparent xylene and sealed with neutral gum.

### Data evaluation

For pyrosequencing, the quality of DNA methylation analysis data was assessed with the Graphpad prism 6 analyzer software. Student’s *t*-test and ANOVA were used for statistical comparisons of DNA methylation levels at *BMPR2* for categorical variables. For qRT-PCR results, paired *t*-test and *χ*^2^ test were used for statistical analysis. The diagnostic value of DNA methylation of *BMPR2* and its expression was evaluated by the receiver operating characteristic (ROC) curve. Data are shown as mean value ± SD, with *P* < 0.05 regarded as statistically significant.

## RESULTS

### *BMPR2* DNA methylation was higher in the VHD-PAH patients

**Figure 1:**
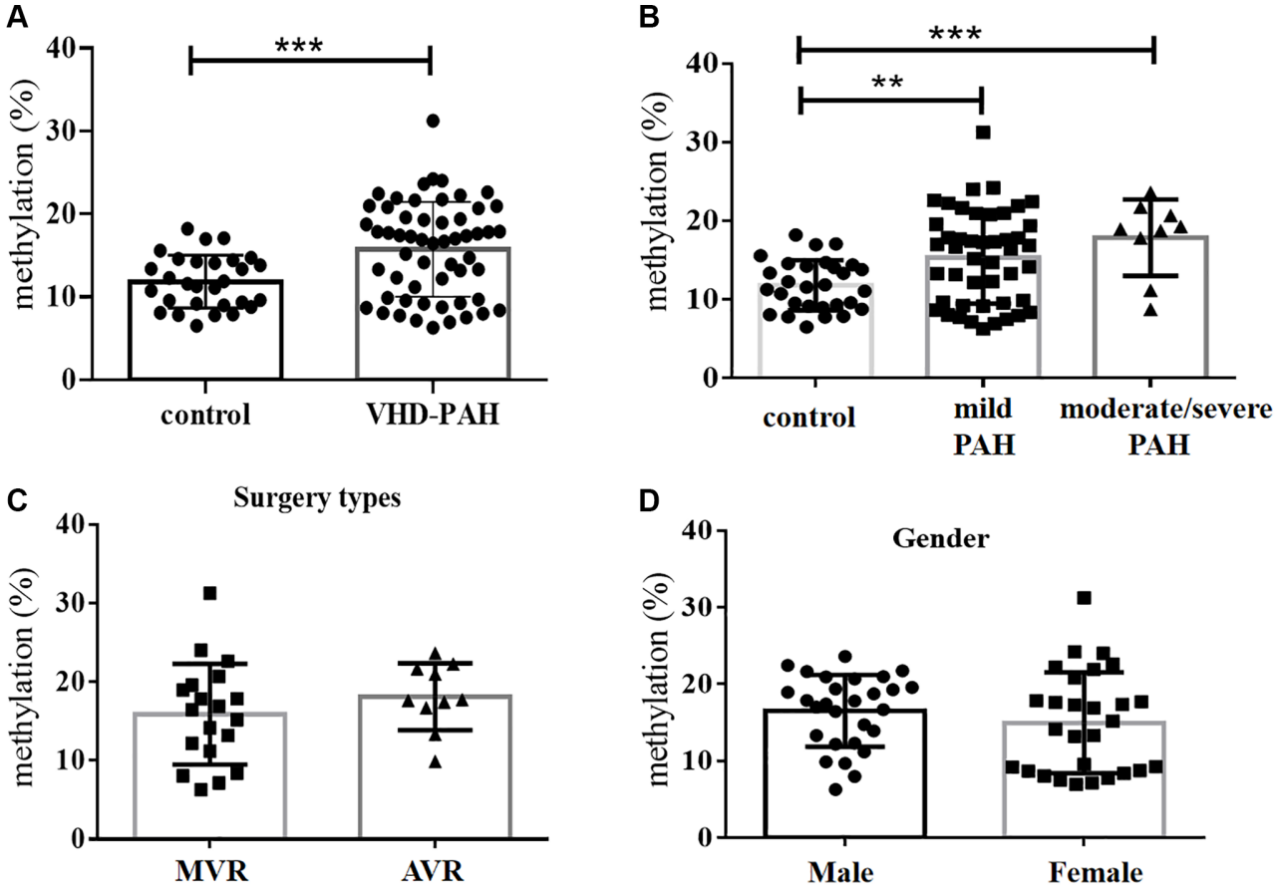
**DNA methylation levels at the *BMPR2* promoter were significantly increased in the VHD group compared with the control group.** (**A**) Average levels of DNA methylation of 10 CpG sites at the *BMPR2* promoter of the VHD group of 55 patients were compared with those of the healthy control group of 28 people. (**B**) The mean *BMPR2* DNA methylation levels of the patient cases with different pulmonary artery pressure (PASP) were significantly increased compared with the controls. Moderate/severe PAH, PASP ≥ 55 mmHg. Mild PAH, 30mmHg ≤ PASP < 55 mmHg. (**C**) The operation type of MVR or AVR had no effect on the *BMPR2* promoter DNA methylation levels. MVR, Mitral valve replacement; AVR, Aortic valve replacement. (**D**) The mean *BMPR2* methylation levels were not different comparing male VHD patients with female VHD patients. For statistical comparisons, ^**^P < 0.01. ^***^P < 0.001.

**Figure 2:**
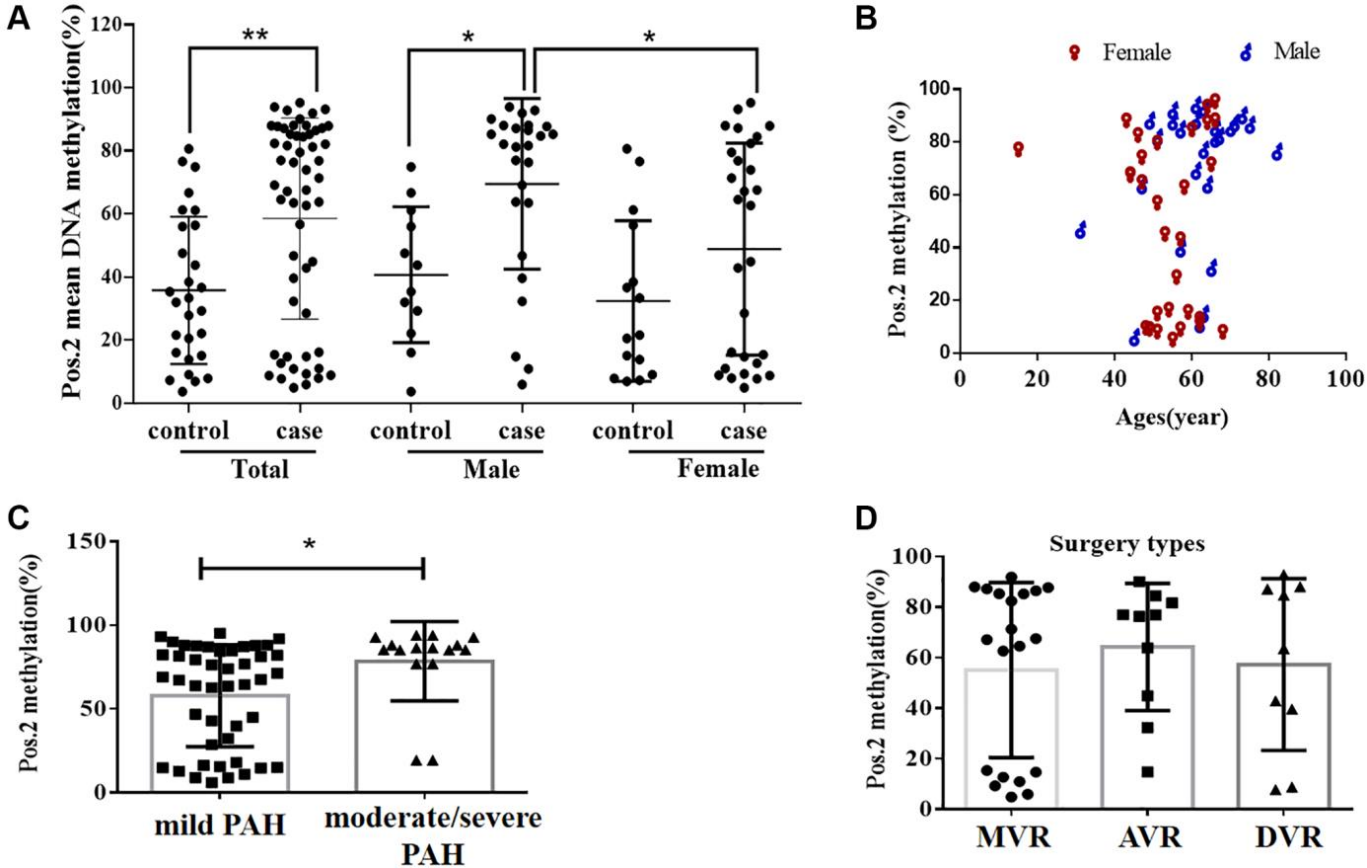
**The DNA methylation level at the second CpG site (Pos. 2) of the *BMPR2* promoter was affected by the VHD clinicopathological factors.** (**A**) The DNA methylation levels at Pos. 2 of *BMPR2* were more significantly increased in the male patients than the females in comparison to the controls. (**B**) The DNA methylation levels at Pos. 2 of *BMPR2* were shown in different age groups of VHD patients. (**C**) The DNA methylation levels at Pos. 2 of *BMPR2* were more significantly increased in the VHD patients with more severe PAH than those with mild PAH. Moderate/severe PAH, PASP≥55mmHg. Mild PAH, 30 mmHg ≤ PASP < 55 mmHg. (**D**) The operation type of MVR or AVR or DVR had no effect on the *BMPR2* promoter DNA methylation levels at Pos. 2 of *BMPR2*. MVR, mitral valve replacement; AVR, aortic valve replacement. DVR, double valve replacement. For statistical comparisons, ^*^P < 0.05. ^**^P < 0.01.

Bisulfite pyrosequencing was performed on a DNA fragment containing the promoter region of *BMPR2*. This fragment contains 10 CpG sites that were measured to evaluate DNA methylation levels at the promoter region of *BMPR2* (Supplementary Figure 1). The mean values of DNA methylation at these 10 CpG sites were used for comparisons between the VHD-PAH group of 55 patients and the healthy control group of 28 people without VHD. We found mean DNA methylation levels at *BMPR2* in the VHD-PAH group were significantly higher (*P* < 0.001) than those in the healthy control group (Figure 1A, Table 2). When these 10 CpG sites at *BMPR2* were analyzed individually, two of them (Pos. 2 and Pos. 9) showed significantly increased DNA methylation in the VHD patients compared with the healthy controls (Table 2, Figure 2A and Supplementary Figure 2A). These results were confirmed in the second cohorts of 27 VHD-PAH patients and 29 healthy control people (see Supplementary Table 1).

**Table 2:** DNA methylation level at 10 CpG sites of *BMPR2* in the control and VHD-PAH patients.

**CpG Position**	**Mean ± SD in the VHD-PAH group (*n* = 55)**	**Mean ± SD in the control group (*n* = 28)**	***P* value**
Pos. 1	4.816% ± 0.976%	4.682% ± 0.7604%	0.9273
Pos. 2	58.58% ± 4.332%	35.78% ± 4.410%	0.0013
Pos. 3	4.298% ± 0.7553%	6.300% ± 1.109%	0.1337
Pos. 4	3.013% ± 0.5665%	1.673% ± 0.6943%	0.1562
Pos. 5	3.439% ± 0.7155%	5.386% ± 1.300%	0.1573
Pos. 6	4.083% ± 0.8052%	5.937% ± 0.9912%	0.1678
Pos. 7	17.68% ± 1.549%	15.02% ± 1.211%	0.2588
Pos. 8	4.837% ± 0.8705%	6.366% ± 1.012%	0.2841
Pos. 9	35.31% ± 2.560%	20.73% ± 2.421%	0.0005
Pos. 10	21.80% ± 2.283%	17.61% ± 1.494%	0.2188
Mean value of 10 CpG sites	15.79% ± 0.7757%	11.83% ± 0.5922%	0.0009

### DNA methylation of *BMPR2* was affected by PASP and gender in VHD patients

We also examined if DNA methylation levels at these CpG sites of *BMPR2* may be affected by the gender, age and PASP levels (Figures 1–2, Supplementary Figure 2). DNA methylation at Pos. 2 or Pos. 9 of *BMPR2* was significantly increased in the male VHD patients that include the VHD patients with MVR, AVR or DVR compared with the controls (Figure 2A, Supplementary Figure 2A). However, DNA methylation at Pos. 9 of *BMPR2* was not significantly increased in the female VHD patients compared with the controls (Figure 2A, Supplementary Figure 2A). Although there was not much difference for overall DNA methylation at *BMPR2* comparing the male VHD patients with the female VHD patients (Figure 1D), we found elevated DNA methylation levels at Pos. 2 of *BMPR2* in the male VHD patients compared with the female VHD patients (Figure 2A). We did not observe any significant difference in DNA methylation at Pos. 2 or Pos. 9 of *BMPR2* in different age groups of the male or female VHD patients (Figure 2B and Supplementary Figure 2B). Except for the slight differences of Pos. 2 and the Pos. 9, there were no gender-specific differences in all VHD-PAH cases in DNA methylation at other 8 positions of *BMPR2* promoter (Supplementary Figure 3).

According to their PASP levels, the VHD patients were divided into two groups: the mild PAH group with 37.81 ± 1.12 mmHg, and the moderate/severe PAH group with 67.00 ± 5.16 mmHg. Based on the pyrosequencing results, higher DNA methylation levels were present at the CpG sites of *BMPR2* in the VHD patients with mild PAH compared with the healthy controls, and even higher DNA methylation levels were observed at the CpG sites of *BMPR2* in the VHD patients with moderate/severe PAH compared with the healthy controls (Figure 1B). When the CpG sites were analyzed individually, DNA methylation was significantly increased at Pos. 2 but not at Pos. 9 at the promoter of *BMPR2* in the VHD group with moderate/severe PAH compared with the VHD group with mild PAH (Figure 2C, Supplementary Figure 2C).

We also tested the effects of different operation types of valve replacement surgery on *BMPR2*. There was no apparent effect of different operation types of valve replacement surgery (MVR, AVR and DVR) on DNA methylation at *BMPR2* when the patient samples derived from these operation types were compared with each other (Figure 1C, Figure 2D, Supplementary Figure 2D). In addition, we assessed the effect of duration period of anti-coagulation drug warfarin on *BMPR2* DNA methylation levels with 1 month (1M) of warfarin treatment after the valve replacement surgery or with warfarin treatment for 2–4 years (2–4Y) after the valve replacement surgery (Supplementary Figure 4). We found slightly higher methylation levels in patients with long-term usage of warfarin anticoagulant for 2–4 years than those who had only used warfarin anticoagulant for 1 month after surgery (Supplementary Figure 4A). In particular, increased DNA methylation was observed at two CpG sites (Pos. 5 and Pos. 6) after 2–4 years of warfarin treatment compared with that after 1 month of warfarin use (Supplementary Figure 4B–4C), suggesting the impact of long-term use of warfarin on *BMPR2* DNA methylation.

For the study group of patients with cardiac valvular disease and pulmonary artery hypertension, the commonly used surgical interventions were divided into two types: mechanical valve replacement plus warfarin and biological valve replacement plus aspirin (Supplementary Figure 4D–4E). DNA methylation at two CpG sites (Pos. 2 and Pos. 9) of the *BMPR2* promoter was significantly higher in the biological valve replacement group with warfarin than that in the mechanical valve replacement group with aspirin. These results suggest that aspirin might have greater effect on *BMPR2* methylation than warfarin (Supplementary Figure 4D–4E). It is also possible that biological valve replacement might have a bigger impact on *BMPR2* methylation than mechanical valve replacement (Supplementary Figure 4D–4E). These can be tested in the future.

### *BMPR2* mRNA were decreased in the VHD cases

**Figure 3:**
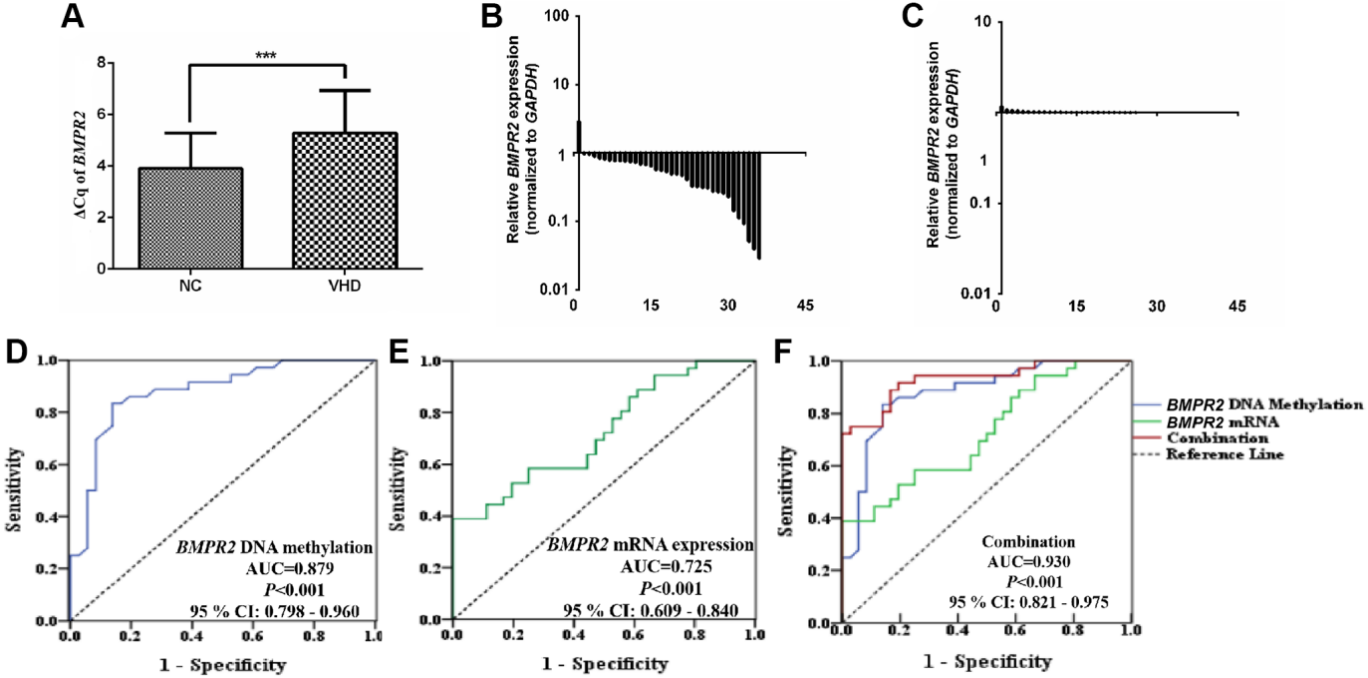
**The *BMPR2* mRNA levels were significantly reduced in the VHD cases compared with the healthy control samples.** Potential diagnostic value was assessed for using the *BMPR2* promoter DNA methylation levels or *BMPR2* mRNA levels or both in the VHD plasma samples. (**A**) The levels of *BMPR2* mRNA were quantified by qRT-PCR in the VHD cases (*n* = 36) and healthy control samples (*n* = 36). ^***^P < 0.001. Since *BMPR2* was not highly expressed, the difference in the Cq values (ΔCq) during qRT-PCR was used instead to compare its expression in the VHD patients with that of the controls (NC). (**B**) The *BMPR2* mRNA levels were reduced in 97.2% (35/36) of the VHD cases compared with the healthy control samples. The *BMPR2* mRNA levels were normalized to those of *GAPDH* in these samples. Horizontal axis, VHD patient number. Vertical axis, relative *BMPR2* mRNA levels based on 2^−ΔΔCq^ after normalization. (**C**) The normalized *BMPR2* mRNA levels in non-VHD healthy control (NC) samples. Horizontal axis, control sample number. Vertical axis, relative *BMPR2* mRNA levels based on 2^−ΔΔCq^ after normalization. (**D**) Receiver operating characteristic curve (ROC) analysis was performed when the mean DNA methylation levels of 10 CpG sites at the *BMPR2* promoter were used to distinguish plasma samples of VHD patients from those of normal controls. *P* < 0.001. AUC, area under curve. (**E**) ROC analysis of the *BMPR2* mRNA levels was performed to distinguish plasma samples of VHD patients from those of normal controls. *P* < 0.001. (**F**) ROC analysis was performed when both mean *BMPR2* promoter DNA methylation and mRNA levels were used in combination to distinguish plasma samples of VHD patients from those of the controls. 95% CI: 95% Confidence Interval. ^***^P < 0.001.

DNA methylation at the promoters usually affects gene expression. Thus, we also quantified the levels of *BMPR2* mRNA in 36 VHD patients and 36 healthy controls by qRT-PCR. To find out if the *BMPR2* mRNA was specifically amplified by qRT-PCR, we analyzed the amplified PCR product which showed a single peak in the melting curve, indicating that the qRT-PCR reaction for *BMPR2* was specific and it did not produce any non-specific product. Compared with the healthy controls, *BMPR2* was significantly downregulated in the VHD patients, with reduced *BMPR2* mRNA level observed in 35 out of 36 VHD patients (Figure 3A–3B). By contrast, *BMPR2* was expressed similarly in the healthy controls (Figure 3C). Besides plasma, heart valve tissue samples of VHD patients after valve replacement were also used to determine DNA methylation of the *BMPR2* promoter and *BMPR2* transcript levels. There were some difficulties and ethical problems in obtaining the heart valve specimens of healthy people. Thus, we collected the samples from the patients with valvular heart disease combined with mitral valve stenosis. They were divided into two different groups based on the severity of pulmonary artery hypertension as described above: one group with 11 moderate/severe PAH patients (55 mmHg ≤ PASP) and the other group with 5 mild PAH patients (30 mmHg ≤ PASP < 55 mmHg) (Supplementary Figure 5). There was no significant difference in *BMPR2* mRNA levels based on qRT-PCR (Supplementary Figure 5). However, this may be due to huge variations in human heart valve tissue samples, which may need to be investigated further.

### Effects of other clinicopathological factors on *BMPR2* mRNA levels

We also examined any association between *BMPR2* mRNA levels and other clinicopathological characteristics of the VHD patients such as age and gender. As described in Table 3, the levels of *BMPR2* mRNA were significantly impacted by the differences in age (*P* = 0.018), PASP (*P* = 0.017), *BNP* (*P* = 0.016), troponin-I (*P* = 0.007) and operation type (*P* = 0.021). It appears that higher PASP, *BNP* and troponin-I in the VHD patients resulted in lower levels of *BMPR2* mRNA (Table 3). However, there was no association between the levels of *BMPR2* mRNA and other clinicopathological factors including gender, smoking, surgical methods, and the duration of warfarin treatment after valve replacement surgery (Table 3). Expression of *BMPR2* was determined by qRT-PCR for the heart valve tissue of 11 human VHD patients with moderate/severe PAH and 5 human VHD patients with mild PAH. There was no significant difference in *BMPR2* transcript, which may be partly due to big variations in human heart valve tissue (Supplementary Figure 5). Higher DNA methylation was observed at one CpG sites (Pos. 9) of the *BMPR2* promoter using the heart valve tissue from the VHD patients after valve replacement with moderate/severe PAH compared with that of human VHD patients with mild PAH (shown in Supplementary Table 2).

**Table 3:** The putative effects of some clinicopathological factors on *BMPR2* expression levels (Δ*C*_q_) in the plasma of the VHD-PAH patients.

**Characteristics**	**Numbers of VHD-PAH patients (%)**	**Numbers of High PAH (%)**	**Numbers of Low PAH (%)**	***P* value (*BMPR2* mRNA)**
All cases	36 (100%)	18 (50%)	18 (50%)	
Gender				0.310
Male	15 (41.7%)	9(50%)	6 (33.3%)	
Female	21 (58.3%)	9(50.0%)	12 (66.7%)	
Age (Years)				0.018
≤ 60	15 (41.7%)	4 (22.2%)	11 (61.1%)	
> 60	21 (58.3%)	14 (77.8%)	7 (38.9%)	
Smoking				0.296
Yes	13 (36.1%)	8 (44.4%)	5 (27.8%)	
No	23 (63.9%)	10(55.6%)	13 (72.2%)	
Surgical Methods				0.477
Mitral valve	11 (30.6%)	5 (27.8%)	6 (33.3%)	
Aortic valve	13 (36.1%)	6 (33.3%)	7 (38.9%)	
Mitral valve & Tricuspid valve	2 (5.6%)	1 (5.55%)	1 (5.6%)	
Mitral valve & Aortic valve	10 (27.7%)	6 (33.35%)	4 (22.2%)	
PASP				0.017
Low	6 (16.7%)	4 (22.2%)	2 (11.1%)	
Mild	22 (61.1%)	7 (38.9%)	15 (83.3%)	
Severe	8 (22.2%)	7 (38.9%)	1 (5.6%)	
*BNP* (ng/L)				0.016
≤ 200	8 (22.2%)	7 (38.9%)	1 (5.6%)	
> 200	28 (77.8%)	11 (61.1%)	17 (94.4%)	
Troponin-I (ng/mL)				0.007
≤ 0.2	9 (25%)	8 (44.4%)	1 (5.6%)	
> 0.2	27 (75%)	10 (55.6%)	17 (94.4%)	
Operation type				0.021
MVR	11 (30.6%)	2 (11.1%)	9 (50%)	
AVR	13 (36.1%)	8 (44.45%)	5 (27.8%)	
DVR	12 (33.3%)	8 (44.45%)	4 (22.2%)	
Warfarin duration				0.422
Within 1 month	8 (22.2%)	3 (16.7%)	5 (27.8%)	
2–4 years (2-4Y)	28 (77.8%)	15 (83.3%)	13 (72.2%)	

### Potential diagnostic value of *BMPR2*

Since VHD patients exhibited higher levels of DNA methylation at the promoter of *BMPR2* and lower levels of *BMPR2* mRNA, we wonder if we may be able to use these values for diagnostic purposes in the VHD cases. Therefore, according to the mRNA expression and methylation rate of *BMPR2* in VHD and normal controls, receiver operating characteristic (ROC) curves of *BMPR2* DNA methylation and mRNA expression were established to test their potential diagnostic values in VHD. The area under the ROC curve (AUC) of *BMPR2* DNA methylation reached 0.879, with 86.1% for sensitivity, 83.3% for specificity, and 95% Confidence Interval (CI) ranging from 0.798 to 0.96 (Figure 3D). When the Youden index is the highest, it corresponds to the best cutoff threshold. Therefore, the cutoff value for *BMPR2* DNA methylation was 13.49 (% methylation). The AUC value of *BMPR2* mRNA level reached 0.725, with 76.1% for sensitivity and 81.5% for specificity, and 95% CI ranging from 0.609 to 0.84 (Figure 3E). The cutoff value of *BMPR2* mRNA level was 6.1 (ΔC_q_). When DNA methylation and *BMPR2* mRNA level were used in combination for ROC, the AUC value increased to 0.930, with 89.8% for sensitivity and 91.9% for specificity, and 95% CI ranging from 0.821 to 0.975 (Figure 3F). Based on these analyses, we think *BMPR2* DNA methylation and mRNA levels can serve as good biomarkers in VHD.

### *Bmpr2* promoter methylation was increased in the lung of a pulmonary artery hypertension (PAH) mouse model

**Figure 4:**
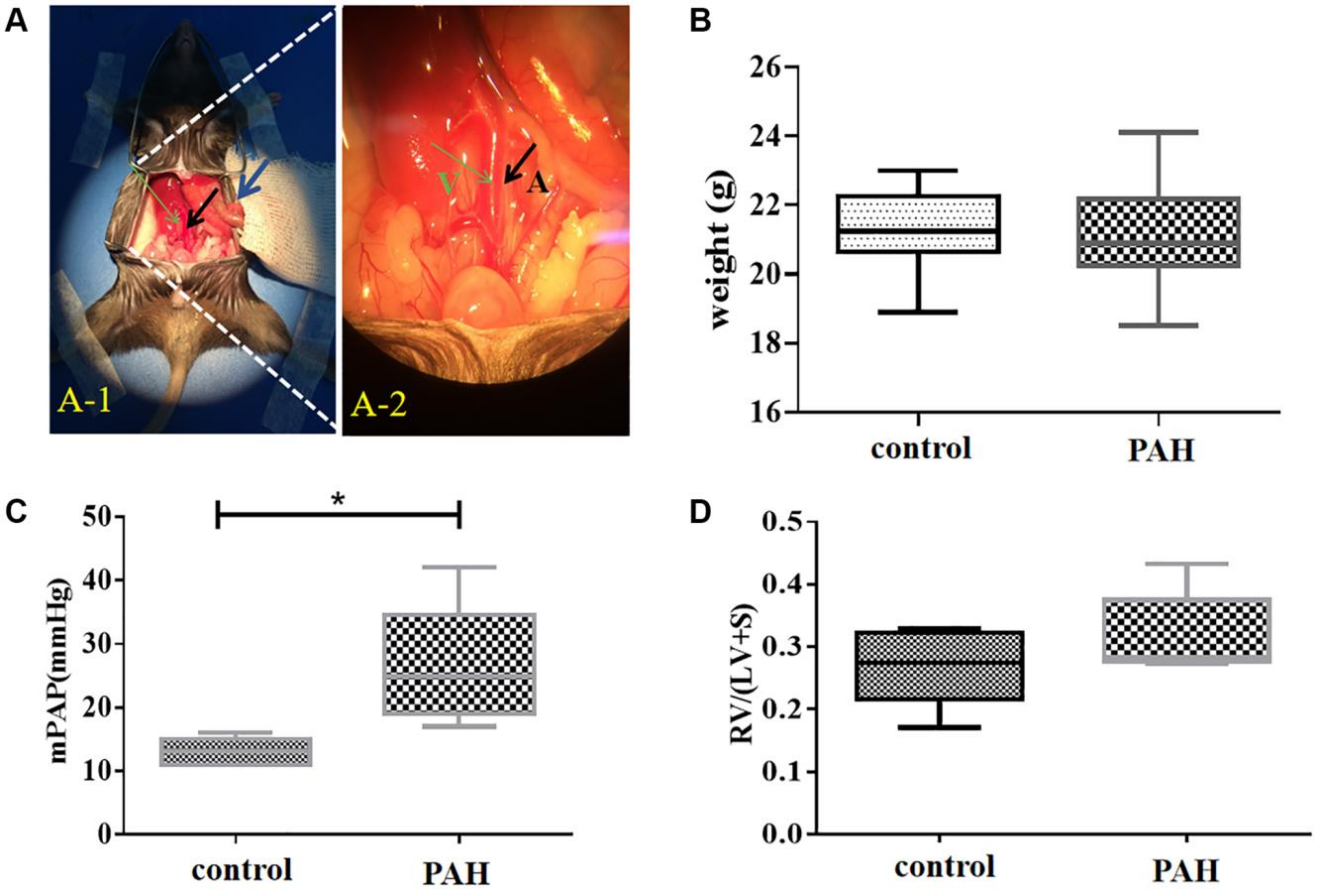
**Shunt-associated pulmonary artery hypertension (PAH) model was established and evaluated in mice with abdominal shunt.** (**A**) A pulmonary artery hypertension (PAH) mouse model was generated by the abdominal shunt technique. (A-1), a mouse was shown for undergoing the surgery to create a shunt-associated pulmonary artery hypertension (PAH) mouse model. The intestine of the mouse intestinal tube (blue arrow) was pushed aside to allow the abdominal aorta (black arrow) and inferior vena cava (thin green arrow) to be exposed for operation. (A-2), a magnified image of the surgical site in (A-1) for the operation to induce the pulmonary artery hypertension. After surgery, red blood flowed past the proximal heart end of the inferior vena cava (V, thin green arrow), indicating the success of the surgery. Black arrow, abdominal aorta (**A**). (**B**) There was not much change in the average mouse weight in the PAH model group compared with that in the control group when they were measured in about 8 weeks after the surgery. (**C**) The pulmonary artery pressure was increased in the PAH mice compared with the control mice when they were examined in 8 weeks after surgery. (**D**) Right ventricular hypertrophy index (RVHI) was measured and compared between the PAH mice and the controls in 8 weeks after surgery. ^*^P < 0.05.

We generated a pulmonary artery hypertension (PAH) mouse model with an abdominal shunt (Figure 4A). The PAH mice had similar weight to the control mice (Figure 4B). But they displayed much higher pulmonary artery pressure than the control mice (Figure 4C). The right ventricular hypertrophy index also appeared to be increased in the PAH mice compared with the control mice although it was not statistically significant (Figure 4D).

### BMPR2 was reduced in the heart and lung of the PAH model mice

**Figure 5:**
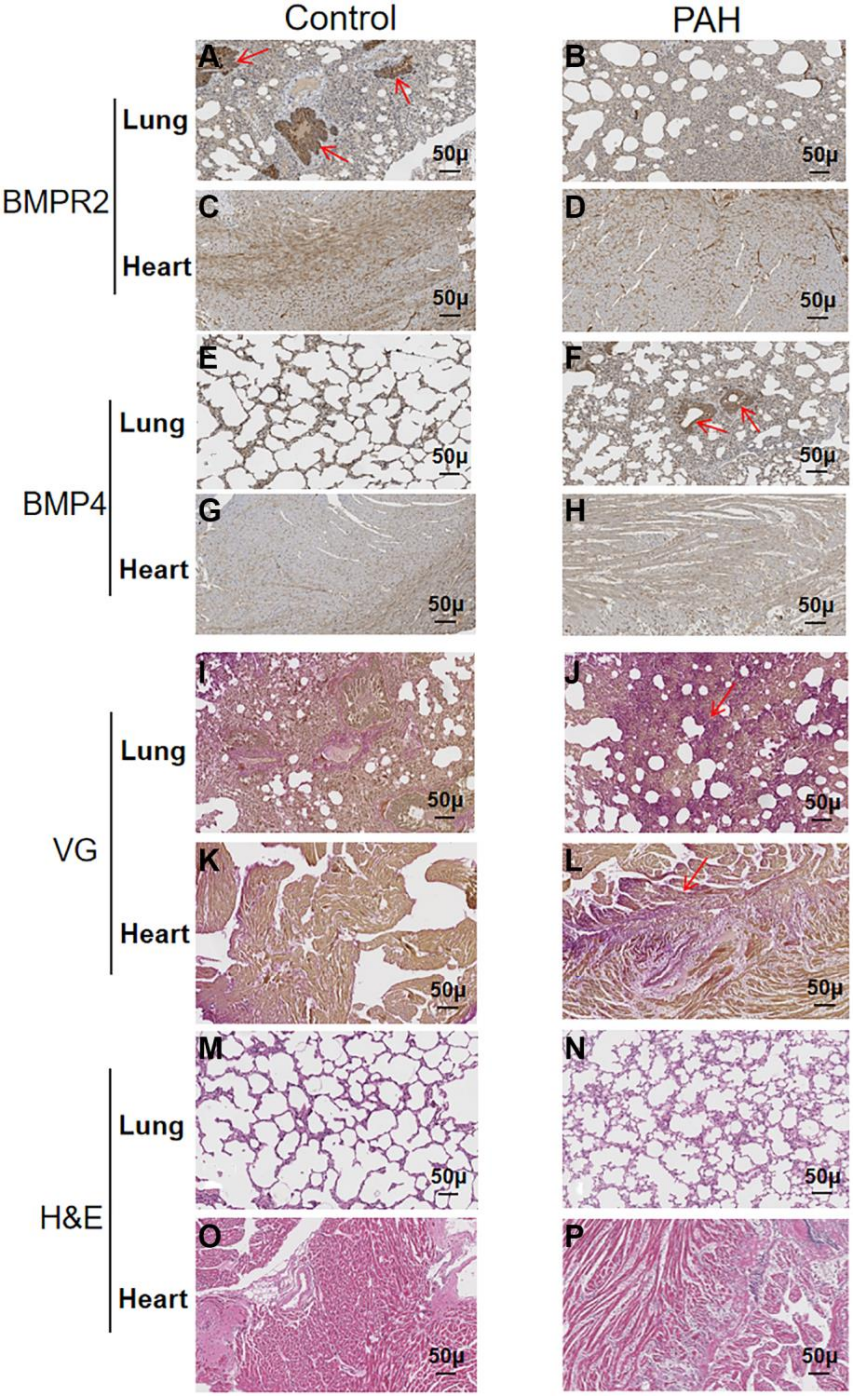
**Reduced BMPR2 expression and higher fibrosis were observed in a pulmonary artery hypertension (PAH) mouse model.** Abdominal shunt was used to create a pulmonary artery hypertension (PAH) mouse model. The lung and heart sections of 4 PAH mice were compared with those of 4 control mice. Scale bars, 50 μm. (**A**–**D**) BMPR2 expression was reduced on the lung section of the pulmonary hypertension model mice compared with that of the control mice based on immunohistochemistry staining. The brown stained areas indicated by the arrows in Figure 5A showed high BMPR2 protein levels in the lung section of the control mice. This reduction of BMPR2 was not observed in the heart sections comparing PAH mice with the control mice. (**E**–**H**) Higher BMP4 expression was observed on the lung section but not on the heart section of the PAH mice compared with the control mice by immunohistochemistry. The brown stained areas indicated by the arrows in Figure 5F showed high BMP4 protein levels on the lung section of the PAH mice. (**I**–**L**) Fibrosis was observed in the PAH mice. Van Gieson staining was carried out for the lung and heart section of the PAH and control mice. The area indicated by the arrow in Figure 5J and Figure 5L showed fibrosis on the lung and heart section of the PAH mice, respectively. (**M**–**P**) hematoxylin-eosin staining was carried out for the lung and heart section of the PAH and control mice.

BMPR2 was highly expressed around the pulmonary vessels of the control mice based on immunohistochemistry (IHC) staining but it was reduced in the lung section of the PAH mice (Figure 5A–5B) (Supplementary Table 3). BMPR2 immunostaining might be also reduced in the heart section of the PAH mice compared with the control mice (Figure 5C–5D) (Supplementary Table 3). By contrast, BMP4 was increased in the pulmonary vascular cells as well as in the heart sections of the PAH mice compared with the control mice although the differences were more obvious in the lungs of the PAH mice versus the control mice (Figure 5E–5H) (Supplementary Table 3). It appeared that BMPR2 signaling pathway may be inhibited in the lung and maybe in the heart as well in the PAH mice. These results are consistent with reduced *BMPR2* expression observed in the PAH patients.

We also performed VG and H&E staining on the lung and heart sections of the PAH mice (Figure 5I–5P) (Supplementary Table 4). Based on VG staining, fibrous hyperplasia was observed on the lung and heart sections of the PAH mice compared with the control mice (Figure 5I–5L) (Supplementary Table 4). These results suggest that impaired BMPR2 signaling pathway may cause fibrosis in the PAH mice resulting from the structural remodeling of heart and lung tissues. These have also been observed in patients with valvular heart disease complicated with pulmonary artery hypertension (VHD-PAH).

**Figure 6:**
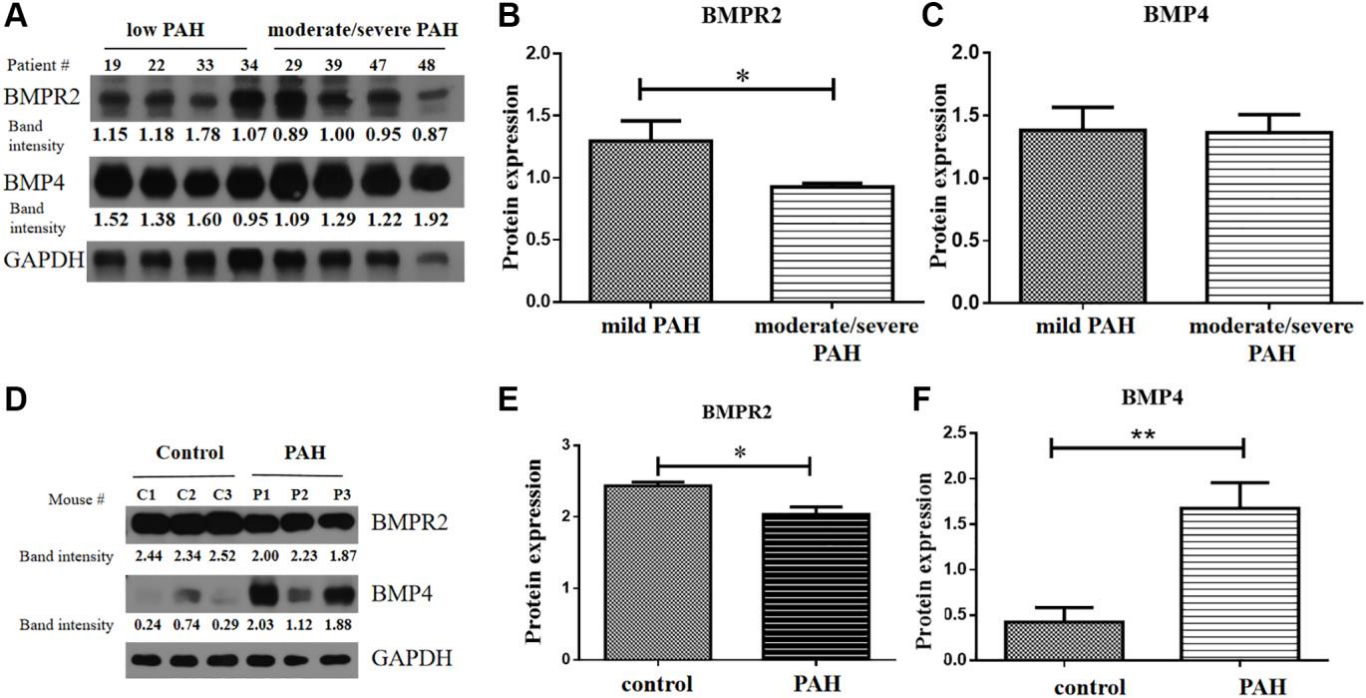
**BMPR2 was downregulated in human PAH patients as well as in the PAH model mice.** (**A**–**C**) Western blot analysis of human patient samples. (**A**) Western blot analysis was carried out to examine BMPR2 and BMP4 protein levels in the heart valve tissues samples derived from the human PAH patients after surgery with mild (30 mmHg ≤ PASP < 55 mmHg) and moderate to severe (PASP ≥ 55 mmHg) pulmonary hypertension. The band intensities of BMPR2 and BMP4 in four low PAH patients (#19, #22, #33, #34) and four moderate/severe PAH patients (#29, #39, #47, #48) were normalized to those of GAPDH on the western blots based with the ImageJ analysis. The numbers are shown for each band of BMPR2 and BMP4 in each patient sample. These normalized protein levels are shown in Figure 6B for BMPR2 and Figure 6C for BMP4. Mild PAH, patients with low PASP. Moderate/severe PAH, patients with moderate to severe PASP. (**D–F**) Western blot analysis of mouse PAH samples. (**D**) Western blot analysis was carried out to examine BMPR2 and BMP4 protein levels in the heart tissues samples derived from the PAH model mice. The band intensities of BMPR2 and BMP4 in three control (C1, C2, C3) and three PAH model mice (P1, P2, P3) were normalized to those of GAPDH on the western blots based with the ImageJ analysis. The numbers are shown for each band of BMPR2 and BMP4 in the PAH model mice. These normalized protein levels are shown in Figure 6E for BMPR2 and Figure 6F for BMP4. Statistical analysis: ^*^P < 0.05. ^**^P < 0.01.

BMPR2 and BMP4 protein levels were tested in the heart tissue samples of the human VHD patients and PAH model mice by western blots. BMPR2 was reduced in heart valve tissue samples of human VHD patients after valve replacement with moderate/severe PAH compared with those of human VHD patients with mild PAH (Figure 6A–6B). But BMP4 appeared to be similar in heart valve tissue samples of human VHD patients after valve replacement with moderate/severe PAH or with mild PAH (Figure 6A, 6C). We do not know how BMPR2 and BMP4 protein levels are in the VHD patients compared with the healthy controls since the heart tissue samples of the normal people were not available for this kind of experiment. BMPR2 was also down-regulated in the heart samples of the PAH mice compared with the control mice, whereas BMP4 was increased in the hearts of the PAH mice versus the control mice (Figure 6D–6F). These results are consistent with the IHC results of the heart and lung samples of the PAH mice (Figure 5A–5H). Taken together, these results suggest that deregulation of BMP signaling may be important for some observed phenotypes in the PAH mice and human VHD-PAH patients.

### Apoptosis was increased in the hearts of the PAH mice

**Figure 7:**
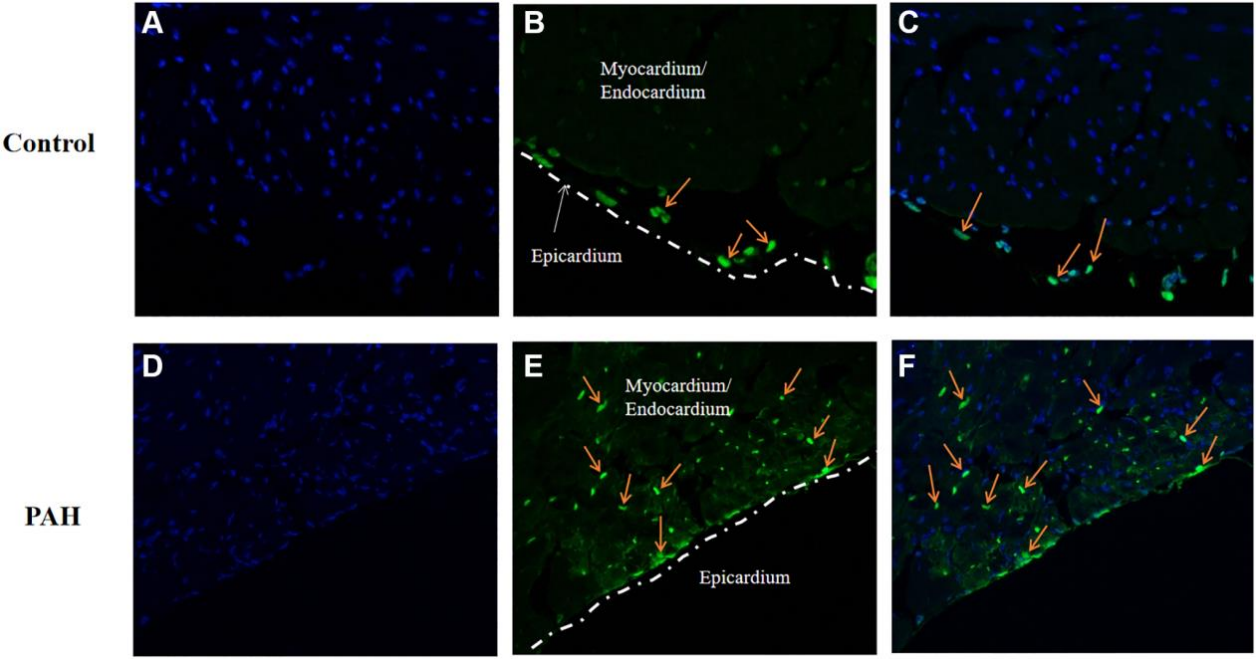
**Apoptosis was increased in the PAH model mice.** TUNEL staining was used to examine apoptotic cells on the heart sections of the control and PAH model mice. (**A**) the image of the DAPI-stained (blue staining) heart section of the control mice. (**B**) the image of the TUNEL-stained (green staining) heart section of the control mice. Arrow, TUNEL-stained apoptotic cell. (**C**) the merged image of DAPI-stained (blue staining) and TUNEL-stained (green staining) heart section of the control mice. Arrow, TUNEL-stained apoptotic cell. (**D**) the image of the DAPI-stained (blue staining) heart section of the PAH mice. (**E**) the image of the TUNEL-stained (green staining) heart section of the PAH mice. Arrow, TUNEL-stained apoptotic cell. (**F**) the merged image of DAPI-stained (blue staining) and TUNEL-stained (green staining) heart section of the PAH mice. Arrow, TUNEL-stained apoptotic cell.

We wonder if apoptosis might be involved in the pathogenesis in VHD-PAH. Hence, we tested expression of the key genes in apoptosis in the PAH mice. Interestingly, apoptosis appeared to be increased in the hearts of the PAH mice. Figure 7A and 7D were images of DAPI stained. TUNEL-positive cells were only found in the epicardium of the control mice (Figure 7B–7C). By contrast, there were a lot of TUNEL-positive cells in the myocardium/endocardium of the PAH mice besides TUNEL-positive cells in the epicardium (Figure 7E–7F). Figure 7A and 7D are namely the images of the DAPI-stained heart section of the control and PAH group as background. Thus, apoptosis may indeed play a role in VHD-PAH.

## DISCUSSION

DNA methylation is an epigenetic modification that is essential for normal development of many organisms. It is involved in X chromosome inactivation, genomic imprinting and retroviral silencing, etc [31–33]. Dysregulation of DNA methylation causes a variety of human diseases. Hypomethylation and hypermethylation has been observed at some genomic locations in the cardiovascular diseases [34, 35]. However, it is not known if DNA methylation may play an important role in VHD. In this study, we found the promoter of *BMPR2* was hypermethylated in VHD-PAH patients in two separate cohort studies (Figures 1–2, Supplementary Figure 2, Supplementary Table 1). Furthermore, *BMPR2* mRNA levels decreased in the VHD-PAH patients compared with healthy controls (Figure 3A–3B). These results are consistent with the previous studies on the relationship between the *BMPR2* promoter methylation and its expression in the VHD patients. Our findings suggest that there is also strong correlation between DNA hypermethylation at the *BMPR2* promoter and the occurrence of the VHD-PAH cases.

In this study, we also examined if we can apply our findings to diagnosis of VHD cases. Indeed, both *BMPR2* promoter DNA methylation and *BMPR2* mRNA levels in the plasma are good biomarkers of VHD, with the AUC of 0.879 for *BMPR2* DNA methylation and the AUC of 0.725 for *BMPR2* mRNA, respectively (Figure 3C–3D). Hypermethylation of *BMPR2* was confirmed in the second cohort study, which is the further proof of its diagnostic value for VHD (Supplementary Table 1). It has been shown that combined diagnosis with multiple biomarkers can increase diagnostic value [36, 37]. We also tested if combination of *BMPR2* promoter DNA methylation and *BMPR2* mRNA levels may be even better in diagnosis of VHD. Indeed, the AUC has increased to 0.930 when both *BMPR2* promoter DNA methylation and *BMPR2* mRNA levels were used in combination (Figure 3E).

Currently, there are some examination methods in use for Rheumatic Valvular Heart Disease (RVHD) including electrocardiography (ECG), chest X-ray, Transthoracic Electrocardiography (TTE), Transesophageal Electrocardiography (TEE) and cardiac catheter tests. A few methods have examination-related complications such as puncture injury, induced adverse reactions, mucosal injury bleeding, laryngeal spasm, mandibular joint dislocation and arrhythmia. The relatively high examination cost is another problem for some methods. Therefore, only the diagnostic values of ECG and TTE are chosen to be compared with those of *BMPR2* developed in this study. ECG and TTE have the respective sensitivity of 0.70 and 0.78, with respective specificity of 0.62 and 0.92 (Supplementary Table 5). Based on our current combined diagnostic method using *BMPR2* promoter methylation and its mRNA expression, the sensitivity and specificity for RVHD is 0.898 and 0.919, respectively (Supplementary Table 5). Therefore, our new method has achieved higher sensitivity than ECG and TTE. Its specificity is equivalent to that of TTE, but is better than that of ECG. In addition, our current diagnostic method is easy to use and its cost is much lower compared with others. Therefore, we believe it can significantly improve diagnosis of VHD, with increased sensitivity and specificity.

Pathogenesis of PAH is influenced by epigenetic factors, genetic polymorphisms, environmental and pathological factors [38]. PAH is often present in clinical complications of VHD. Reduction in the area of the pulmonary vasculature in the late stages of PAH can often lead to heart failure or right ventricular hypertrophy [39]. Some studies have shown that DNA methylation may be involved in PAH and VHD [40–42]. It is known that *BMPR2* has important functions in the cardiovascular system. It has also been associated with the increased risk in VHD. *BMPR2* mutations were found to be present in 55%−70% of high pulmonary arterial hypertension (HPH) cases and 11%−40% of idiopathic pulmonary arterial hypertension (IPH) cases [43]. *BMPR2* is involved in growth, differentiation and apoptosis of epithelial and mesenchymal cells during pulmonary vascular smooth muscle hypertrophy and pulmonary vascular remodeling, which results in pulmonary hypertension [21, 44]. DNA methylation at the promoters usually regulates expression of the target genes. Since we observed decreased expression of *BMPR2* in the VHD patients with increased methylation at *BMPR2*, our results imply that hypermethylation at *BMPR2* may cause VHD through down-regulation of *BMPR2* expression. This gives additional support to the diagnostic values of our current methods.

Valve replacement therapy is a main treatment option for the patients with VHD-PAH after they are admitted into the hospital [45, 46]. Different surgical methods may result in different lesions in mitral, aortic or double valves, which may also partly reflect the heart function of VHD patients. However, we did not find any significant differences in *BMPR2* promoter DNA methylation levels when MVR, AVR or DVR was performed for the VHD patients (Figure 2D). It was not influenced by age either. We also found long-term warfarin anticoagulation therapy slightly affect the *BMPR2* promoter DNA methylation in VHD patients undergoing mechanical heart valve replacement. Interestingly, DNA methylation levels at Pos. 2 of *BMPR2* seemed to be more significantly increased in the male VHD patients than the female VHD patients in comparison to the healthy controls (Figure 2A). This suggests that the male patients may be predisposed to VHD, similar to what were reported in other studies [47–49].

In contrast, we found *BMPR2* mRNA level was statistically significantly affected by age (*P* = 0.018), PASP (*P* = 0.017), *BNP* (*P* = 0.016), troponin-I (*P* = 0.007) and operation type (*P* = 0.021). PASP can also influence the DNA methylation levels of *BMPR2*. This suggests that we may be able to predict PASP based on DNA methylation of the *BMPR2* promotor and *BMPR2* mRNA levels. The condition of PASP can be used to improve the diagnosis efficiency besides *BMPR2*. *BNP* and troponin-I are indicative of heart failure and myocardial ischemic necrosis in heart patients, respectively [50]. *BNP* or troponin-I may be also used in combination with *BMPR2* for better diagnosis.

Consistent with what we found in human VHD patients, we found BMPR2 was significantly down-regulated in the lung and heart of PAH model mice, whereas BMP4 was increased in the lung and heart of PAH model mice (Figures 5–6). DNA methylation was increased at the *Bmpr2* promoter in the lung of the PAH model mice. Furthermore, we observed increased fibrosis in the lung and heart of PAH mice (Figure 5). These results suggest that deregulation of BMP signaling may play a role in VHD-PAH.

There are some limitations to our current study that may be improved in the future. First, only a fragment containing 10 CpG sites of the *BMPR2* promoter was used for DNA methylation analysis in this study. We should try to find out in the future if there are additional CpG sites of *BMPR2* that may also have good diagnostic values. We should also test if there are other additional factors that may affect DNA methylation of *BMPR2* in VHD. Third, we should assess the potential functions of histone modifications at the *BMPR2* gene in VHD. Finally, there is some difficulty in generating the mouse model of VHD-PAH. We may need to create a better VHD mouse model to further study the underlying mechanisms of BMPR2 in VHD-PAH pathogenesis.

## Supplementary Materials

Supplementary Figures

Supplementary Tables
