# Supplementary material for: BMPR2 promoter methylation and its expression in valvular heart disease complicated with pulmonary artery hypertension
Source: Aging (Albany NY). 2021 Nov 18;13(22):24580–604. doi: 10.18632/aging.203690 (PMC8660616; doi:10.18632/aging.203690)
Supplement: Supplementary Figures [file aging-13-203690-s001.pdf]

SUPPLEMENTARY FIGURES

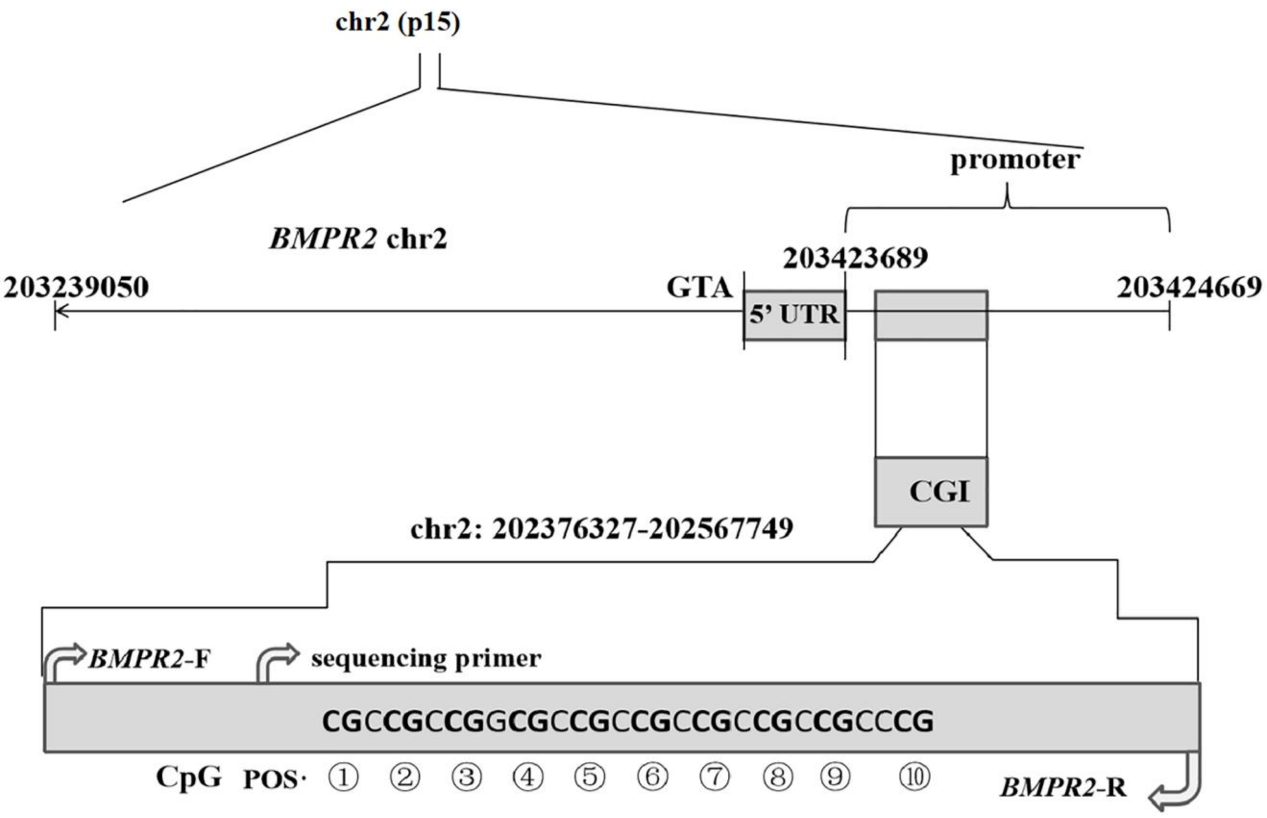

Supplementary Figure 1. Diagram is shown for the PCR primer information and the 10 CpG sites (1–10) at the promoter of the human *BMPR2* gene that were used for DNA methylation analysis. The human *BMPR2* gene is located on Chromosome 2 (Chr. 2). CGI, CpG island. The positions of the primers used for PCR or sequencing are marked in this diagram.

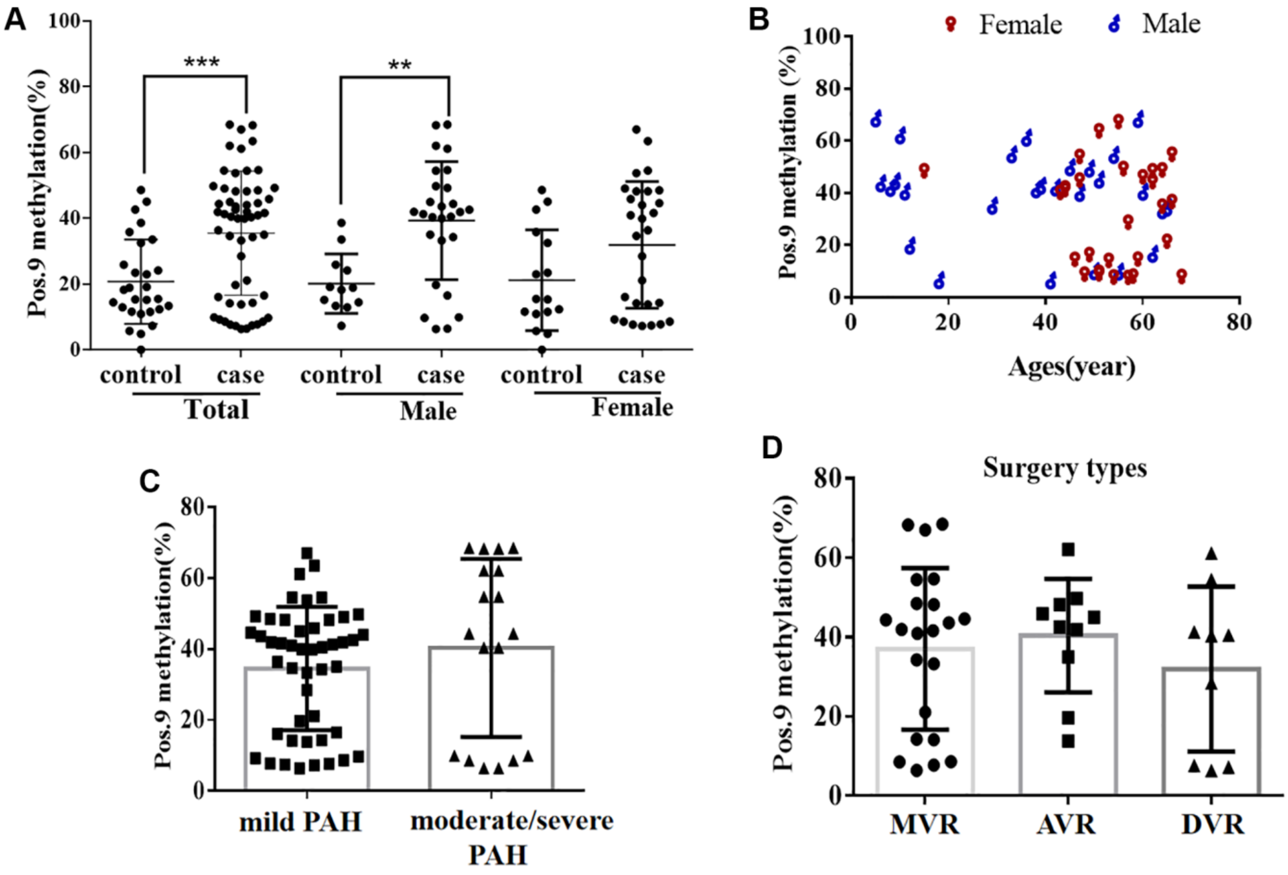

Supplementary Figure 2. The DNA methylation level at the ninth CpG site (Pos. 9) of the *BMPR2* promoter was affected by the VHD clinicopathological factors. (**A**) The DNA methylation levels at Pos. 9 of *BMPR2* were more significantly increased in the male patients than the females in comparison to the controls. (**B**) The DNA methylation levels at Pos. 9 of *BMPR2* in different age groups of VHD patients. (**C**) The DNA methylation levels at Pos. 9 of *BMPR2* in different age groups of VHD patients with different PASP. Moderate/severe PAH, PASP ≥ 55 mmHg. Mild PAH, 30 mmHg ≤ PASP < 55 mmHg. (**D**) The operation type of MVR or AVR or DVR had no effect on the *BMPR2* promoter DNA methylation levels at Pos. 9 of *BMPR2*. MVR, mitral valve replacement; AVR, aortic valve replacement. DVR, double valve replacement. For statistical comparisons, $^{**}P < 0.01$. $^{***}P < 0.001$.

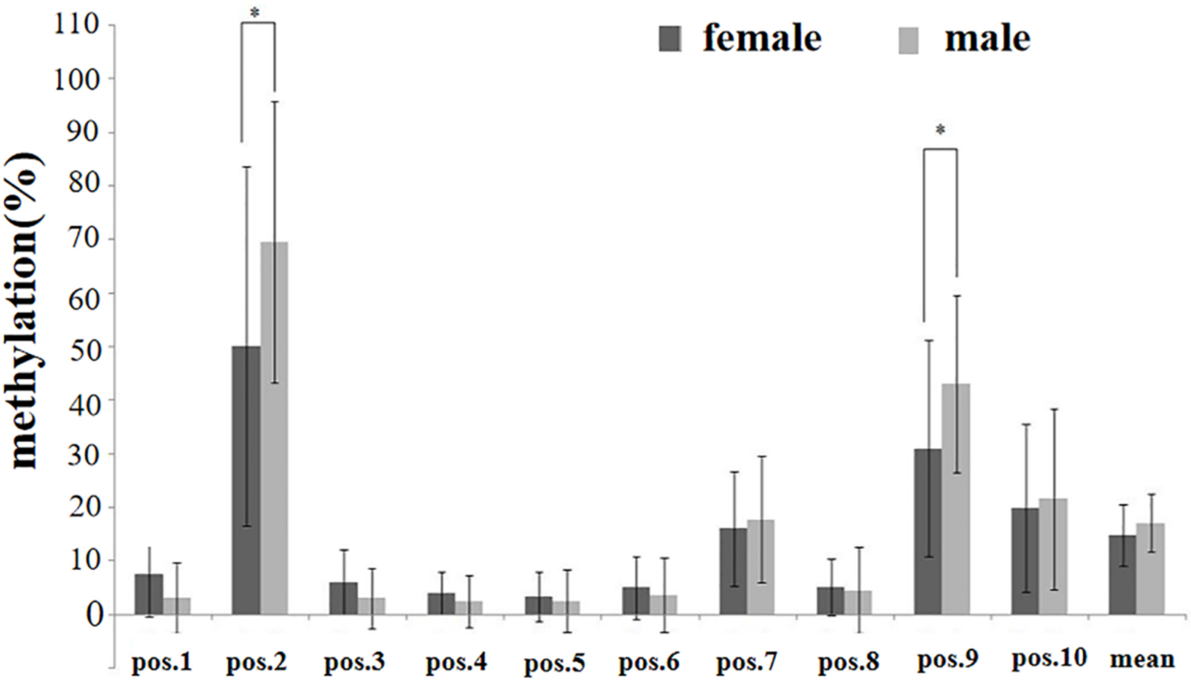

Supplementary Figure 3. Gender had little effect on DNA methylation at most CpG sites of the *BMPR2* promoter in the VHD-PAH patients. DNA methylation at 10 CpG sites (Pos. 1–10) of the *BMPR2* promoter was assessed for the male and female patients with the valvular heart disease complicated with pulmonary hypertension (VHD-PAH). DNA methylation at each of 10 CpG sites (Pos. 1–10) of the *BMPR2* promoter was assessed for any difference in the samples of the male and female VHD-PAH patients. Only two CpG sites (Pos. 2 and Pos. 9) of the *BMPR2* promoter exhibited statistically significant differences in DNA methylation comparing male VHD-PAH patients with female VHD-PAH patients, with slightly more methylated in the male patients. Statistical analysis: $^{*}P < 0.05$.

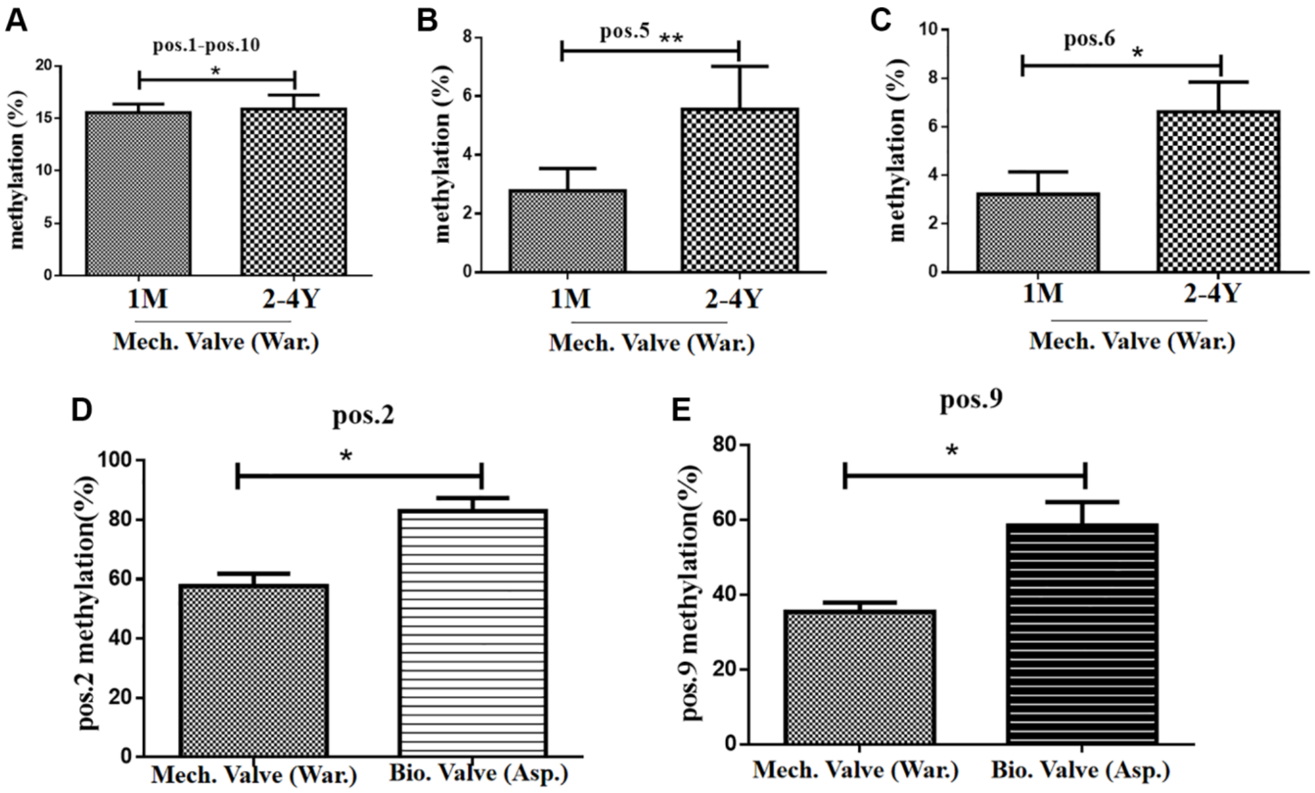

Supplementary Figure 4. The DNA methylation levels of *BMPR2* was assessed after application of the anticoagulant and hemorrhage drug warfarin in the patients. All 10 CpG sites (Pos. 1–10) of the *BMPR2* promoter were assessed for the DNA methylation levels by the valvular heart disease complicated with pulmonary hypertension (VHD-PAH) patients with different warfarin dose requirements after valve replacement. 1M, taking warfarin (War.) treatment for 1 month after mechanical valve (Mech. Valve) replacement. 2-4Y, taking warfarin (War.) treatment after mechanical valve (Mech. Valve) replacement for 2–4 years. (**A**) Mean methylation levels of Pos. 1–10 of the *BMPR2* promoter. (**B**) Methylation levels of Pos. 5 of the *BMPR2* promoter. (**C**) Methylation levels of Pos. 6 of the *BMPR2* promoter (**D**) Methylation levels of Pos. 2 of the *BMPR2* promoter after mechanical valve (Mech. Valve) replacement with warfarin (War.) or biological valve (Bio. Valve) replacement with aspirin (Asp.). (**E**) Methylation levels of Pos. 9 of the *BMPR2* promoter after mechanical valve (Mech. Valve) replacement with warfarin (War.) or biological valve (Bio. Valve) replacement with aspirin (Asp.). Statistical analysis: $^{*}P < 0.05$. $^{**}P < 0.01$.

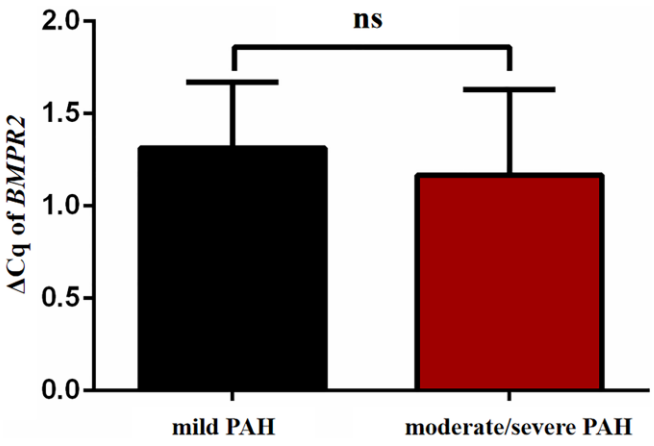

Supplementary Figure 5. There was no difference in *BMPR2* mRNA levels of the human heart valve tissue samples of the mild PAH and moderate/severe PAH patients. *BMPR2* mRNA was quantified by qRT-PCR for the human heart valve tissue samples derived from the mild PAH and moderate/severe PAH patients. Since *BMPR2* transcript levels were too low, the difference in the Cq values (ΔCq) during qRT-PCR was used instead to compare its expression in the mild PAH patients with that of moderate/severe PAH patients. ns, not statistically significant.
