# Supplementary material for: BMPR2 promoter methylation and its expression in valvular heart disease complicated with pulmonary artery hypertension
Source: Aging (Albany NY). 2021 Nov 18;13(22):24580–604. doi: 10.18632/aging.203690 (PMC8660616; doi:10.18632/aging.203690)
Supplement: Supplementary Tables [file aging-13-203690-s002.pdf]

## SUPPLEMENTARY TABLES

**Supplementary Table 1. DNA methylation level at 10 CpG sites of *BMPR2* in the second cohorts of VHD-PAH patients and healthy controls.**

| CpG Position | Mean ± SD in the VHD-PAH group ($n$ = 27) | Mean ± SD in the control group ($n$ = 29) | $P$ value |
|---|---|---|---|
| Pos. 1 | 3.396% ± 0.4164% | 1.239% ± 0.5329% | 0.0871 |
| Pos. 2 | 50.05% ± 3.821% | 29.75% ± 2.932% | 0.0059 |
| Pos. 3 | 4.623% ± 0.7612% | 4.381% ± 0.6114% | 0.8065 |
| Pos. 4 | 2.479% ± 0.4316% | 1.536% ± 0.4238% | 0.1311 |
| Pos. 5 | 3.866% ± 0.8075% | 3.941% ± 0.6784% | 0.9436 |
| Pos. 6 | 5.790% ± 1.037% | 3.437% ± 0.5867% | 0.0880 |
| Pos. 7 | 15.39% ± 1.433% | 12.91% ± 0.3509% | 0.1510 |
| Pos. 8 | 6.5395% ± 1.167% | 4.828% ± 0.6793% | 0.2172 |
| Pos. 9 | 29.06% ± 2.322% | 17.65% ± 1.655% | 0.0001 |
| Pos. 10 | 20.73% ± 2.099% | 15.05% ± 1.107% | 0.3470 |
| Mean value of 10 CpG sites | 13.16% ± 0.7635% | 8.743% ± 0.5544% | 0.0009 |

**Supplementary Table 2. DNA methylation of 10 CpG sites of the *BMPR2* promoter in the cardiac valve specimens of the PAH patients.**

| Methylation (%) | Pos. 1 | Pos. 2 | Pos. 3 | Pos. 4 | Pos. 5 | Pos. 6 | Pos. 7 | Pos. 8 | Pos. 9 | Pos. 10 | Mean |
|---|---|---|---|---|---|---|---|---|---|---|---|
| Mild PAH | 5.75 ± 1.44 | 5.67 ± 1.18 | 8.59 ± 3.40 | 6.87 ± 0.80 | 8.44 ± 0.94 | 8.08 ± 0.77 | 8.23 ± 0.62 | 8.00 ± 0.40 | 6.68 ± 1.39 | 8.35 ± 0.77 | 7.47 ± 0.77 |
| Moderate/ Severe PAH | 7.56 ± 1.65 | 7.39 ± 1.87 | 8.72 ± 2.60 | 8.36 ± 2.15 | 9.57 ± 1.67 | 9.86 ± 2.36 | 10.30 ± 2.45 | 9.83 ± 2.41 | 8.32 ± 1.35* | 10.12 ± 1.81 | 8.96 ± 1.86 |
| $p$ | 0.07 | 0.06 | 0.93 | 0.17 | 0.17 | 0.08 | 0.09 | 0.08 | 0.04 | 0.07 | 0.09 |

**Supplementary Table 3. BMPR2 and BMP4 protein expression in the human heart valve tissue samples based on immunohistochemistry (IHC).**

| IHC for protein expression | Tissue | Groups | Percentage contribution of High Positive & Positive (%) | Percentage contribution of Low Positive (%) | Percentage contribution of Negative (%) |
|---|---|---|---|---|---|
| BMPR2 | Lung | control | 14.74 ± 0.7813* | 40.33 ± 0.4954 | 44.94 ± 1.145 |
| | | PAH | 10.02 ± 1.552 | 34.71 ± 3.308 | 55.27 ± 4.199* |
| | Heart | control | 8.509 ± 1.520 | 56.20 ± 4.989 | 35.29 ± 6.186 |
| | | PAH | 5.784 ± 0.3746 | 50.53 ± 4.655 | 43.69 ± 4.622 |
| BMP4 | Lung | control | 12.16 ± 0.6811 | 22.42 ± 5.482 | 66.28 ± 8.868 |
| | | PAH | 15.12 ± 0.8344* | 35.88 ± 1.279* | 53.70 ± 5.432 |
| | Heart | control | 2.600 ± 0.3343 | 48.92 ± 3.002 | 46.51 ± 3.385 |
| | | PAH | 1.644 ± 0.3046 | 46.57 ± 0.881 | 51.79 ± 0.7491 |

*$P < 0.05$.

**Supplementary Table 4. Quantification of fibrosis based on VG and HE staining in the heart and lung of the PAH model mice.**

| Groups | VG staining | Area fraction % | HE staining | Area fraction % |
|---|---|---|---|---|
| control | Lung | 25.48 ± 1.861* | Lung | 16.08 ± 3.322 |
| PAH | | 32.27 ± 2.644 | | 14.21 ± 3.144 |
| control | Heart | 14.89 ± 0.3421 | Heart | 10.90 ± 2.115 |
| PAH | | 16.24 ± 4.228 | | 10.86 ± 0.6797 |

*$P < 0.05$.

**Supplementary Table 5. Comparisons of sensitivity and specificity for our methods and others in VHD-PAH diagnosis.**

| VHD group VS. control group | sensitivity | specificity |
|---|---|---|
| *BMPR2* mRNA | 0.761 | 0.815 |
| *BMPR2* DNA methylation | 0.861 | 0.833 |
| combination | 0.898 | 0.919 |
| ECG | 0.70 | 0.62 |
| TTE | 0.78 | 0.92 |
